# Education, sense of mastery and mental health: results from a nation wide health monitoring study in Norway

**DOI:** 10.1186/1471-244X-7-20

**Published:** 2007-05-22

**Authors:** Odd Steffen Dalgard, Arnstein Mykletun, Marit Rognerud, Rune Johansen, Per Henrik Zahl

**Affiliations:** 1 Norwegian Institute of Public Health, Division of Mental Health, P.O Box 4404 Nydalen, 0403 Oslo, Norway

## Abstract

**Background:**

Earlier studies have shown that people with low level of education have increased rates of mental health problems. The aim of the present study is to investigate the association between level of education and psychological distress, and to explore to which extent the association is mediated by sense of mastery, and social variables like social support, negative life events, household income, employment and marital status.

**Methods:**

The data for the study were obtained from the Level of Living Survey conducted by Statistics Norway in 2002. Data on psychological distress and psychosocial variables were gathered by a self-administered questionnaire, whereas socio-demographic data were based on register statistics. Psychological distress was measured by Hopkins Symptom Checklist 25 items.

**Results:**

There was a significant association between low level of education and psychological distress in both genders, the association being strongest in women aged 55–67 years. Low level of education was also significantly associated with low sense of mastery, low social support, many negative life events (only in men), low household income and unemployment,. Sense of mastery emerged as a strong mediating variable between level of education and psychological distress, whereas the other variables played a minor role when adjusting for sense of mastery.

**Conclusion:**

Low sense of mastery seems to account for much of the association between low educational level and psychological distress, and should be an important target in mental health promotion for groups with low level of education.

## Background

Mental health problems have become a major public health concern, epidemiological studies showing that up to one-fifth or one-quarter of the general population suffer from some sort of mental disorder at a given time [1-3]. For this reason, it is of great public health interest to monitor the development in various countries, with respect to morbidity as well as factors that might influence mental health.

For the purpose of monitoring mental health in the population, the EU has recently recommended a set of indicators [4]. The set is developed as a project under the EC Health Monitoring programme, and is based on the collection of information on existing mental health indicators at both national and international levels, and the review of literature. The following domains are covered by the indicators: Socio-demographic, social networks, stressful life events, positive mental health (i.e. sense of mastery), quality of life, services (supply, use and demand), morbidity (generic), morbidity (disease specific), disability and mortality. Some of this information may be collected from registers and existing statistics, but most of the information has to be collected by special surveys. This variety of indicators could be used not only to describe mental health in terms of morbidity and use of health services, but also to identify individual and environmental factors which influence mental health, in a positive or negative way. A number of the mental health indicators and measuring instruments suggested by the European Union have been adopted in the Norwegian Health and Level of Living Survey. This nation wide survey takes place every three year, and is conducted by Statistics Norway. In the present paper some of the recommended mental health indicators and measuring instruments will be used to investigate the relationship between education and mental health.

There are various mechanisms which might explain the association between poor mental health and low education. Selection could be one of them, suggesting that children and adolescents with poor mental health, or with increased vulnerability because of individual and/or environmental factors, will be less able to complete higher education. Low education may also contribute to poor mental health through poor economy and problems on the labour market. Lastly, low educational level could be associated with a low sense of mastery, which could be a stressor in itself, or indirectly influence mental health through other psychosocial variables, like social support [5].

Higher rates of psychological distress in people with little education have been reported in several studies [6,7]. There are also studies reporting an association between perceived lack of control and health problems, somatic as well as mental [8-11], and studies reporting that lack of control is an important mediator between socio-economic status and somatic health [12,13]. One study reports perceived control to be among the strongest mediators between level of education and psychological wellbeing [14], and another that lack of control at work is an important mediator between employment grade and depressive symptoms [15]. In a recent survey paper Marmot (2006) suggests that lack of control or autonomy is among the most important mediators between socioeconomic status and health [16].

The aim of the present study was to explore to which extent sense of mastery mediates the effect of education on mental health, when adjusting for the effect of socio-demographic factors, social support and negative life events

## Methods

### Sample

The data in the present study were obtained from the Health and Level of Living Survey conducted by Statistics Norway in 2002, covering 10, 000 individuals above the age of 15 years. The data on mental health and psychosocial variables were obtained by postal questionnaire, after an initial interview by home visit or telephone. Data on education, income and marital status were based on register statistics from Statistics Norway. The response rate was 70.4%. Non-response analysis showed that the non-responders differed only slightly from the total sample with respect to gender, age and place of living in Norway. Only those in the age group 25–67 years were included in present the study, and the final sample with data on education counted 4446 persons. Missing data on other social and psychosocial variables were substituted by means.

### Variables

#### Mental health

Mental health was measured by the HSCL-25 [17] which consists of 25 questions about symptoms of depression, anxiety and common psychosomatic symptoms during the last 14 days. Each question was scored on a scale from 1 (not bothered) to 4 (extremely bothered), and the HSCL-score was calculated as the sum score of items divided by number of items answered. Only cases with response to more than 20 items were included. Missing data were substituted with mean values of missing items. The Cronbach's alpha of the scale in the present sample was 0.94.

#### Sense of mastery

Sense of mastery is measured by a 5-item version of a 7-items scale developed by Pearlin et al [6] comprising the following items:

There is really no way I can solve some of the problems I have

Sometimes I feel that I'm pushed around in life

I have little control over the things that happen to me

I often feel helpless in dealing with the problems of life

There is little I can do to change many of the important things in my life

The responses (strongly disagree, disagree, disagree as much as agree, agree, strongly agree), were numbered from 1 to 5, and summarised into the score of sense of mastery. The Cronbach's alpha for the scale in the sample was 0.86.

The correlation between sense of mastery and psychological distress was high (Pearson correlation 0.6), and one may ask to which extent the two concepts are overlapping. To investigate this, principal component analysis (Oblimin with Kaiser Normalization) was carried out. The result is shown in Table 1.

**Table 1:** Principal component analysis of the items in Sense of mastery and HSCL-25 Rotation method: Oblimin with Kaiser Normalization

Two factor extraction Items	Components	
	
	1	2
Cannot solve problems	-,374	,768
Pushed around in life	-,462	,805
Little control	-,372	,803
Often feel helpless	-,586	,796
Little to do to change life	-,419	,732
		
Headaches	,350	-,240
Tremble	,511	-,170
Faintness/dizziness	,579	-,337
Nervous/shaky	,787	-,383
Suddenly scared	,715	-,229
Fearful	,767	-,311
Heart pound	,563	-,213
Tense/keyed up	,678	-,345
Spells/terror	,699	-.218
Restless	,518	-,176
Low energy	,657	-,483
Blame self	,663	-,432
Cry easily	,554	-,332
Thoughts of ending life	,576	-,216
Poor appetite	,533	-,212
Difficulty sleep	,567	-,373
Hopeless	,753	-,532
Blue	,794	-,484
Lonely	,694	-,418
Loss of sex pleasure	,507	-,383
Feeling trapped	,555	-,327
Worry to much	,785	-,452
Feel no interest	,693	-,409
Everything effort	,720	-,527
Worthlessness	,663	-,463

**Figure 1:**
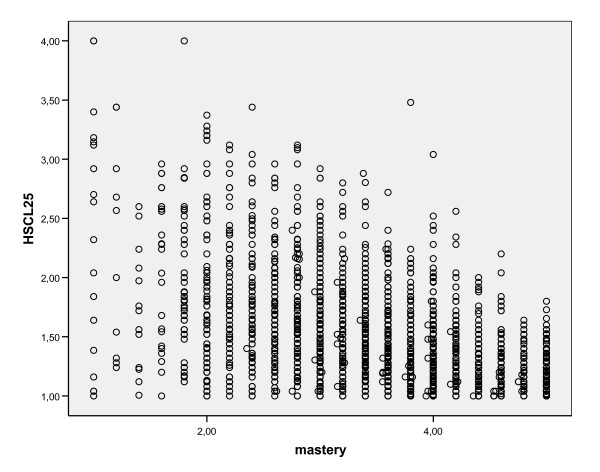
Scatterplot HSCL-25 against sense of mastery.

Two component extraction resulted in components with loadings between 0.7 and 0.8 (sense of mastery) and between 0.5 and 0.7 (HSCL-25), with the exception of one item, *headache*, which loaded 0.4 on the HSCL-25 component. Explained variance by the first component was 39.6% and by the second component 6.5%. Three and four component extractions resulted in a split of the first component in respectively two and three components, first anxiety/depression and somatization, and then anxiety, somatization and depression, which are known as subcomponents of HSCL-25. The second component, sense of mastery, remained unchanged under three and four component extraction. From this we conclude that sense of mastery is a factor different from HSCL-25 and its subcomponents. Also a scatter plot (Figure 1) supported the suggestion that we are dealing with two separate factors.

Whereas those with strong sense of mastery were likely to have low psychological distress, low sense of mastery only to a limited extent predicted high psychological distress.

#### Social support

The Oslo social support scale [18,19] was used to measure social support. This scale consists of three items concerning number of confidants, the feeling of interest and concern from others, and the possibility for practical help from neighbours. The z-scores of each item are summarized into a total score of social support.

#### Negative life events

Negative life events were measured by a 12 item scale concerning major negative life events during the last 12 months [20].

#### Socio-demographic variables

Education was split in three groups according to years of education: 7–10 years (low), 11–14 years (moderate) and 15 years or more (high). Net household income was defined as the sum of net income in the household divided by the square root of household members, and split in eight ordinal categories. Marital status was dichotomized into "married/cohabitant" and "others", and employment status was dichotomized into "usually in paid work" and "others". "Usually in paid work" was defined as having at least one hour of paid work during the last week (including work in family enterprise or farm), or having been temporarily away from paid work last week. Hence, "others" is a complex group, including unemployed, people on disability pension, home-makers and students.

### Statistical methods

For a mediator to be demonstrated, besides an association between the initial and the outcome condition, the possible mediator has to be associated with both conditions. In this case, level of education has to be associated with psychological distress, and sense of mastery and the other possible mediators to both these variables. To investigate this, and to explore the mediating effects, the following analyses have been carried out:

The association between education and psychological distress was tested by linear regression analysis, across gender and age groups. Possible interactions were looked into by multivariate linear regression analyses.

The associations between level of education and sense of mastery, social support, negative life events, household income and paid work were tested by Pearson correlation, and between level of education and marital status by chi square statistics.

The associations between sense of mastery, the other independent variables and psychological distress were tested by multivariate linear regression analyses across gender, first adjusting for age and then for all independent variables.

In the final analysis, the mediating effects of sense of mastery and other variables on the association between level of education and psychological distress were tested by a number of multiple regression analyses, adjusting for various independent factors.

## Results

There was a significant association between level of education and psychological distress across age groups and gender, with the exception of the eldest age group in men (Table 2).

**Table 2:** Linear regression. Associations between level of education and psychological distress (HSCL-25), by gender and age groups

Gender	Age	Unstandardized beta coefficients	Standardized beta coefficients	Significance
Men	25–34 years	-0.12	-0.20	P < 0.001
	35–44 years	-0.06	-0.10	P = 0.015
	45–54 years	-0.08	-0,13	P = 0.001
	55–67 years	-0.03	-0.06	P = 0.136

Women	22–34 years	-0.07	-0.11	P = 0.011
	35–44 years	-0.08	-0.12	P = 0.002
	45–54 years	-0.09	-0.16	P < 0.001
	55–67 years	-0.11	-0.20	P < 0.001

A test did not yield a statistically significant trend, and only the eldest women differed significantly from the rest, showing the highest association between level of education and psychological distress.

The associations between level of education and psychosocial and socio-demographic variables showed an approximately linear trend, with the exception of marital status. The associations in terms of correlations are shown in Table 3.

**Table 3:** Associations between level of education and psychosocial and socio-demographic variables

Gender	Psychosocial and socio-demographic variables	Pearson Correlation	Sign.
Men	Sense of mastery	0.13	P < 0.001
	Social support	0.10	P < 0.001
	Negative life events	-0.07	P = 0.001
	Household income	0.26	P < 0.001
	Not paid work	-0.19	P < 0.001

Women	Sense of mastery	0.17	P < 0.001
	Social support	0.19	P < 0.001
	Negative life events	-0.03	P = 0.146
	Household income	0.25	P < 0,001
	Not paid work	-0.25	P < 0.001

Educational level was positively correlated with sense of mastery, social support, household income and having paid work, and negatively correlated with negative life events (significant only in men). With respect to marital status, men with high or low level of education were more often married than those with moderate level of education (66% in the two first groups against 60% in the last (P = 0.031)). Different from this, women with lowest level of education were more often married than those with moderate or high level of education (67% in the first group against 63% and 58% in the two last groups (P = 0.032)).

We also studied if the differences in correlation coefficients between men and women were associated with different regression slopes. A statistically significant difference was only found for social support and paid work, indicating that these variables were higher associated with level of education in women than in men.

The associations between the psychosocial and socio-demographic variables and psychological distress are shown in Table 4.

**Table 4:** Multiple linear regression. Associations between psychosocial, socio-demographic variables and psychological distress (HSCL-25), by gender

		Standardized beta coefficients			
		
		Adjusted for age	Sign.	Adjusted for all variables	Sign.
Men	Sense of mastery	-0.62	P < 0.001	-0.54	P < 0.001
	Social support	-0.19	P < 0.001	-0.06	P = 0.001
	Negative life events	0.32	P < 0.001	0.16	P < 0.001
	Household income	-0.24	P < 0.001	-0.06	P = 0.001
	Not paid work	0.25	P < 0.001	0.07	P < 0.001
	Marital status	-0.08	P < 0.001	0.00	P = 0.936

Women	Sense of mastery	-0.61	P < 0.001	-0.51	P < 0.001
	Social support	-0.22	P < 0.001	-0.08	P < 0.001
	Negative life events	0.36	P < 0.001	0.19	P < 0.001
	Household income	-0.17	P < 0.001	-0.03	P = 0.120
	Not paid work	0.26	P < 0.001	0.10	P < 0.001
	Marital status	-0.11	P < 0.001	-0.05	P = 0.003

When only adjusting for age, all variables were significantly associated with psychological distress in both genders. Interaction analysis showed that the association between household income and psychological distress was significantly higher in men than in women. For the other variables there were no significant gender differences. The associations remained statistically significant in the fully adjusted model, with the exceptions of marital status in men and household income in women. The strongest association was between sense of mastery and psychological distress, and this association was only slightly reduced when adjusting for the other variables. The associations between psychological distress and the other variables were, however, strongly reduced when adjusting for sense of mastery. This pattern was more or less the same when splitting in four age groups (see additional files 1, 2, 3, 4), sense of mastery explaining most of the variance in psychological distress in all age groups.

The associations between level of education and psychological distress, when adjusting for sense of mastery and the other variables are shown in Table 5.

**Table 5:** Multiple regression. Associations between level of education and psychological distress (HSCL-25) when adjusting for age, sense of mastery and other variables, by gender

		Standardized beta coefficients						
		
		Mod 1	Mod 2	Mod 3	Mod 4	Mod 5	Mod 6	Mod 7
M	Education	-0.12***	-0.05***	-0.05***	-0.04***	-0.04***	-0.02	-0.01
	Mastery		-0.62***	-0.62***	-0.60***	-0.56***	-0.55***	-0.54***
	Mar. status			-0.04***	-0.04***	-0.03	0.00	0.00
	Soc.supp.				-0.07***	-0.07***	-0.06***	-0.06**
	Neg.l.events					0.17***	0.16***	0.15***
	H.h.income						-0.08***	-0.06**
	Not p. work							0.07***
	
	R square	0.01	0.39	0.39	0.39	0.42	0.42	0.42

W	Education	-0.16***	-0.09***	-0.09***	-0.07***	-0.07***	-0.06***	-0.05**
	Mastery		-0.60***	-0.60***	-0.58***	-0.53***	-0.52***	-0.51***
	Mar. status			-0.09***	-0.08***	-0.06***	-0.05**	-0.05**
	Soc.supp.				-0.09***	-0.08***	-0.08***	-0.08***
	Neg.l.events					0.20***	0.20***	0.19***
	H.h.income						-0.04	-0.02
	Not p. work							0.10***
	
	R square	0.03	0.37	0.37	0.38	0.42	0.42	0.43

P < 0.05 *

P < 0.01 **

P < 0.001 ***

Model 1: Adjusted for age

Model 2: Adjusted for age and sense of mastery

Model 3: Adjusted for age, sense of mastery and marital status

Model 4: Adjusted for age, sense of mastery, marital status and social support

Model 5: Adjusted for age, sense of mastery, marital status, social support and negative life events

Model 6: Adjusted for age, sense of mastery, marital status, social support, negative life events and household income

Model 7: Adjusted for age, sense of mastery, marital status, social support, negative life events, household income and paid work

Model 1 shows that level of education was significantly associated with psychological distress in both genders, when adjusting for age. When also adjusting for sense of mastery (model 2), the association between education and psychological distress was strongly reduced in both genders, but it was still significant. Sense of mastery accounted for 58% of the association between educational level and mental health problems in men, and 44% in women. Further adjustment for marital status (model 3) had no effect on the association between level of education and psychological distress in any gender, whereas social support (model 4) had a slight effect in both genders. Negative life events (model 5) had no effect on the association between level of education and psychological distress in any gender, whereas household income and not having paid work had a slight effect (models 6 and 7). When introducing the independent variables one by one in the sequence shown in Table 5, all variables but household income in women, contribute to a statistically significant increase in explained variance of the dependent variable.

In the full model (model 7) the association between level of education and psychological distress is eliminated in men, and strongly reduced in women. Sense of mastery emerges as the most important variable in both genders, with negative life events as next. It is interesting that marital status in men is not longer associated with psychological distress when adjusting for social support, negative life events and household income, which is not the case in women. Interaction analysis shows a significant gender difference only for negative life events, indicating that such events are closer related to psychological distress in women than in men.

## Discussion

There was a significant association between level of education and psychological distress in both genders, those with low education reporting most symptoms of distress. This association was especially strong in women in the age group 55–67 years.

Sense of mastery accounted for about half of the association between level of education and psychological distress, whereas the other variables contributed less to explain the association.

It is a strength of the study that it is based on a an extensive nation wide sample, matching well the population with respect to age, gender and place of living in Norway, and with register based data on socio-demographic variables (education, income and marital status). The response rate, 70%, is somewhat low, and this may to some extent have influenced the results. Even if there may be some under-representation of people with mental and social problems, which is likely to be the case in such studies, this should not to any substantial degree invalidate the internal comparison of groups, which is the focus of the present study.

As a measure of income, one may question the use of net income, as it is known that many of real high income earners have a low net income. This might reduce the association between household income and psychological distress in the study, but it is difficult to say how it would effect the association between sense of mastery and psychological distress. Anyhow, it is not likely that this will substantially influence the main results of the study.

In the context of the present study, it is a weakness that we are dealing with cross-sectional data, which makes it difficult to draw conclusions about causality.

The finding that the association between level of education and psychological distress is strongest in women above the age of 55 years, is somewhat surprising. A possible explanation could be that women in this age group, when their eventual children are grown up, and their role in the family context is less central, will have problems when entering the labour marked if they are lacking in education. In men the problems with lacking education is likely to be stronger in the younger age groups, when problems on the labour market is difficult to combine with the establishment of a family.

The finding that sense of mastery emerged as an important mediating factor between education and psychological distress is in accordance with other studies suggesting that lack of control plays an important role in explaining the association between social inequality and mental health [21-23]. It leaves open, however, two questions: Why is level of education associated with sense of mastery, and why is sense of mastery associated with psychological distress?

One possibility is that the association between sense of mastery and level of education is explained by psychological selection: Because of lower coping skills, which might be explained by genetic and/or early environmental factors, those with low sense of mastery are less likely to succeed in the school system, and end up with little formal education [21].

Another possibility is that low sense of mastery is a reflection of the life situation of people with low education and low social status. Without education the possibility to control one's life situation is reduced, not least with respect to options on the labour marked. Hence environmental factors may influence the sense of mastery [24]. Obviously, factors of psychological selection and factors of environment may play together, and re-enforce each other in positive or negative feed-back circles. The present study, however, does not allow for drawing conclusions about the causation of sense of mastery.

Why is sense of mastery associated with psychological distress? One possibility is that low sense of mastery in itself is a stressor [21,25]. Another possibility is that low sense of mastery is associated with other stressors, like unemployment, low income, weak social support and negative life events, and hence has an indirect effect on mental health. The last possibility, however, seems unlikely in the present study, as the association between sense of mastery and psychological distress was only slightly reduced when adjusting for the other variables. So the study seems to support the hypothesis that low sense of mastery is a stressor in itself.

Because of the strong association between sense of mastery and psychological distress, the possibility of overlapping concepts has been raised. The principal component analysis, suggesting that we are dealing with two separate factors, weakens this hypothesis. Also a scatter-plot points in the direction of two different factors.

## Conclusion

Low level of education is an important risk factor for mental health, and should be kept in mind in psychiatric prevention and mental health promotion. It is likely to be a mental health gain in reducing school dropouts among young people, and in providing support and guidance for those who have educational problems. It is also important to provide education for elder people who enter the labour marked at a later stage in life, most of them women. Since low sense of mastery seems to contribute strongly to the increased rate of mental health problems in those with little education, education should have a strong element of empowerment. School should not only be a place for teaching of theoretical and practical skills, but should also contribute to psychological growth, with the strengthening of coping and mastery as important elements. Education should also be flexible in relation to each pupil's abilities and psychological resources, and practical as well as theoretical assets should be taken into consideration.

The study illustrates how a national health monitoring system may be used in exploring risk factors for mental health, thereby giving ideas for mental health promotion. Repeated surveys every third year, which is the plan in Norway, will strongly strengthen the potentials of this type of health monitoring. At present a reform in the Norwegian school system is launched, aiming at reducing the problems with school drop outs, and with stronger emphasize on practical education and individualized and supportive career planning. It will be interesting to see in the years to come, by repeated surveys, if this affects the relationship between level of education and mental health, and if it contributes to stronger sense of mastery and better mental health of the population.

## Competing interests

The author(s) declare that they have no competing interests.

## Authors' contributions

OSD has conceived the study, done the statistical analysis and written the manuscript.

AM and PHZ have assisted in the statistical analysis and RJ has prepared the data file. All co-authors have contributed to the design of the study, drafted the manuscript and given their final approval of the version to be published.

## Pre-publication history

The pre-publication history for this paper can be accessed here:



## Supplementary Material

Additional File 1 Associations between psychosocial, socio-demographic variables and psychological distress. Age group 25–34 years Click here for file

Additional File 2 Associations between psychosocial, socio-demographic variables and psychological distress. Age group 35–44 years Click here for file

Additional File 3 Associations between psychosocial, socio-demographic variables and psychological distress. Age group 45–54 years Click here for file

Additional File 4 Associations between psychosocial, socio-demographic variables and psychological distress. Age group 55–67 years Click here for file
