# Supplementary material for: Education, sense of mastery and mental health: results from a nation wide health monitoring study in Norway
Source: BMC Psychiatry. 2007 May 22;7:20. doi: 10.1186/1471-244X-7-20 (PMC1887526; doi:10.1186/1471-244X-7-20)
Supplement: Additional File 1 — Associations between psychosocial, socio-demographic variables and psychological distress. Age group 25–34 years [file 1471-244X-7-20-S1.doc]

Additional file 1

|  | | Standardized beta coefficients | |
| --- | --- | --- | --- |
|  | | Adjusted for all variables | Significance |
| Men | Sense of mastery  Social support  Negative life events  H.h.income  Not paid work  Marital status | -0.58  -0.09  0.05  -0.12  0.08  -0.01 | p<0.001  p=0.025  p=0.193  p=0.005  p=0.045  p=0.875 |
| Women | Sense of mastery  Social support  Negative life events  H.h.income  Not paid work  Marital status | -0.50  -0.03  0.26  0.02  0.06  -0.11 | p<0.001  p=0.365  p<0.001  p=0.664  p=0.126  p=0.007 |
