# Supplementary material for: Education, sense of mastery and mental health: results from a nation wide health monitoring study in Norway
Source: BMC Psychiatry. 2007 May 22;7:20. doi: 10.1186/1471-244X-7-20 (PMC1887526; doi:10.1186/1471-244X-7-20)
Supplement: Additional File 2 — Associations between psychosocial, socio-demographic variables and psychological distress. Age group 35–44 years [file 1471-244X-7-20-S2.doc]

Additional file 2

|  | | Standardized beta coefficients | |
| --- | --- | --- | --- |
|  | | Adjusted for all variables | Significance |
| Men | Sense of mastery  Social support  Negative life events  H.h.income  Not paid work  Marital status | -0.46  -0.05  0.03  -0.09  0.08  0.03 | p<0.001  p=0.218  p=0.493  p=0.058  p=0.054  p=0.480 |
| Women | Sense of mastery  Social support  Negative life events  H.h.income  Not paid work  Marital status | -0.53  -0.11  0.17  -0.07  0.06  -0.02 | p<0.001  p=0.001  p<0.001  p=0.053  p=0.085  p=0.535 |
