# Supplementary material for: Education, sense of mastery and mental health: results from a nation wide health monitoring study in Norway
Source: BMC Psychiatry. 2007 May 22;7:20. doi: 10.1186/1471-244X-7-20 (PMC1887526; doi:10.1186/1471-244X-7-20)
Supplement: Additional File 3 — Associations between psychosocial, socio-demographic variables and psychological distress. Age group 45–54 years [file 1471-244X-7-20-S3.doc]

Additional file 3

|  | | Standardized beta coefficients | |
| --- | --- | --- | --- |
|  | | Adjusted for all variables | Significance |
| Men | Sense of mastery  Social support  Negative life events  H.h.income  Not paid work  Marital status | -0.48  -0.09  0.25  -0.05  0.07  -0.01 | p<0.001  p=0.008  p<0.001  p=0.195  p=0.050  p=0.726 |
| Women | Sense of mastery  Social support  Negative life events  H.h.income  Not paid work  Marital status | -0.49  -0.12  0.19  -0.03  0.18  -0.07 | p<0.001  p=0.001  p<0.001  p=0.436  p<0.001  p=0.102 |
