# Supplementary material for: Education, sense of mastery and mental health: results from a nation wide health monitoring study in Norway
Source: BMC Psychiatry. 2007 May 22;7:20. doi: 10.1186/1471-244X-7-20 (PMC1887526; doi:10.1186/1471-244X-7-20)
Supplement: Additional File 4 — Associations between psychosocial, socio-demographic variables and psychological distress. Age group 55–67 years [file 1471-244X-7-20-S4.doc]

Additional file 4

|  | | Standardized beta coefficients | |
| --- | --- | --- | --- |
|  | | Adjusted for all variables | Significance |
| Men | Sense of mastery  Social support  Negative life events  H.h.income  Not paid work  Marital status | -0.59  -0.08  0.10  0.05  0.10  -0.03 | p<0.001  p=0.030  p=0.005  p=0.254  p=0.009  p=0.444 |
| Women | Sense of mastery  Social support  Negative life events  H.h.income  Not paid work  Marital status | -0.49  -0.12  0.19  -0.03  0.18  -0.07 | p<0.001  p=0.089  p<0.001  p=0.120  p<0.001  P=0.460 |
